# Analysis of Human Gut Microbiota Enzymes for Biotechnological and Food Industrial Applications

**DOI:** 10.3390/foods14101794

**Published:** 2025-05-18

**Authors:** Alfonso Torres-Sánchez, Gracia Luque, Pilar Ortiz, Alicia Ruiz-Rodríguez, Ana López-Moreno, Margarita Aguilera

**Affiliations:** 1 Department of Microbiology, Faculty of Pharmacy, Campus of Cartuja, University of Granada, 18071 Granada, Spain; alfonsotorress@correo.ugr.es (A.T.-S.); gracialuque@ugr.es (G.L.); piortiz@ugr.es (P.O.); aliruizrodriguez@ugr.es (A.R.-R.); maguiler@ugr.es (M.A.); 2 Institute of Nutrition and Food Technology “José Mataix” (INYTA), Centre of Biomedical Research, University of Granada, 18016 Granada, Spain; 3 Instituto de Investigación Biosanitaria (IBS), 18012 Granada, Spain

**Keywords:** food enzymes, amylases, cellulases, inulinases, lipases/esterases, nucleases, proteases, gelatinases, laccase, relative enzymatic activity

## Abstract

The human gut microbiota is a complex and dynamic ecosystem, recognized for its valuable and wide array of physiological functions. This study investigated the human gut microbiota as a source of enzymes for innovative applications in the biomedicine, bioremediation, and food and feed biotechnological industries by integrating data from combined in silico and in vitro approaches. A total of 93 easily cultivable strains were selected from a bank of isolated microorganisms generated from the gut microbiota of children under different media and conditions. First, genomic data screening and enzyme interrogation of reference genomes corresponding to the selected species were carried out using a custom bioinformatic searching protocol. The extraction and interpretation of encoding enzymes from the genomic taxa results focused on four major phyla (Bacillota, Bacteroidota, Actinomycetota, and Pseudomonadota) and seven genera (*Bacillus*, *Bacteroides*, *Clostridium*, *Enterobacter*, *Enterococcus*, *Microbacterium*, and *Staphylococcus*) according to their cultivability and biotechnological relevance and interest. A total of 364 enzymes were identified across protein annotations, highlighting amylases, cellulases, inulinases, lipases, proteases, and laccases. Second, phenotypic assays confirmed these main enzymatic activities in 80.6% of 93 isolates. Notable findings included *Bacillus* species displaying relevant amylase and laccase activity. This study demonstrates the utility of combining genomic annotations with functional assays, offering a robust approach for exploiting gut microbiota enzymes to develop innovative and sustainable biotechnological processes. Moreover, regulatory mechanisms governing enzyme expression in gut resilient microbes are essential steps toward unlocking the full potential of gut microbiota-derived biocatalysts.

## 1. Introduction

The human gut microbiota emerged as a transformative and highly influential area of scientific research, with direct implications for health and disease management. It encompasses a diverse array of microorganisms, including bacteria, archaea, fungi, and viruses, which colonize the gastrointestinal tract, collectively contributing to a dynamic and multifaceted ecosystem [1]. The intricate interplay between host and microbiota exemplifies a symbiotic relationship that has co-evolved over millions of years, highlighting its essential role in maintaining nutritional and physiological homeostasis and shaping the immune system [2]. Beyond its traditional roles in nutrition, digestion, metabolism, and immune modulation, the microbiota is now being explored as an untapped reservoir of active enzymes with novel functionalities that may extend well beyond its conventional biological context [3]. These discoveries deepen our understanding of microbial ecosystems and highlight their potential for industrial, medical, and environmental applications.

This innovative perspective emphasizes that gut microbiota should be considered a unique and valuable ecological niche, rich with the potential for new products and discoveries. Researchers are currently exploring its vast diversity, which can lead to the development of innovative and sustainable alternatives to traditional sources of environmental microbial resources [4]. The extraordinary variety of enzymes produced by these microbes is still not fully understood, but it stems from their adaptation to the host body and the nutrient-rich environment of the gastrointestinal tract. This diversity could offer remarkable opportunities for applications in medicine, food, and feed industries, positioning the gut microbiota as a key resource for cutting-edge bio-technological innovations. In essence, the reactions of the gut microbiota are increasingly being recognized as a treasure trove for biological, food, clinical, and bioremediation potential that could revolutionize various fields through new discoveries [5].

These microorganisms produce an expansive repertoire of enzymes capable of de-grading a wide array of substrates, including polysaccharides [6], proteins [7], and li-pids [8], many of which are indigestible by host metabolism. For example, bacterial glycoside hydrolases and polysaccharide lyases enable the degradation of dietary fibers, releasing metabolites such as short-chain fatty acids that contribute to individual health [9]. Furthermore, gut microbiota-derived proteases and lipases contribute to the metabolism of dietary proteins and lipids, respectively, enhancing nutrient assimilation [10]. This enzymatic repertoire and its encoding genes have become key targets for food and clinical research aimed at identifying novel catalysts with unique functionalities that may serve as blueprints for innovative biotechnological applications.

Beyond their physiological roles, gut-derived bacterial enzymes hold considerable potential for food and biomedical applications. Their high substrate specificity, catalytic efficiency, and stability under diverse conditions make them invaluable candidates for various processes, including food processing, pharmaceuticals, and novel bioengi-neering. In the food industry, these enzymes can enhance nutrient bioavailability, im-prove texture, and contribute to the development of functional foods tailored for spe-cific health benefits. In addition, their potential to synthesize bioactive compounds presents promising avenues for drug discovery and therapeutic development [11]. Moreover, these enzymes are increasingly recognized for their role in sustainable bio-technological processes. Their ability to operate under mild, environmentally friendly conditions offers a safer alternative to conventional chemical catalysts, with applica-tions spanning biosolutions, biodegradable material production, and waste manage-ment [12]. The adaptability and efficiency of gut microbiota enzymes could also be harnessed for emerging technologies, such as the development of biofuels, bioplastics, and novel biomaterials, positioning them as crucial contributors to the growing global bioeconomy.

Advances in metagenomics, transcriptomics, and proteomics, combined with high-throughput functional screening, have accelerated the discovery of novel enzymes from microbiota samples [13]. These methods uncover enzymes previously inaccessible through culture-based techniques, expanding the pool of potential biocatalysts for industry. Research into their biochemical properties, structural mechanisms, and regulatory pathways will deepen understanding of their functional diversity and optimize them for industrial and biotechnological applications [14]. Despite extensive genomic and computational data highlighting the theoretical capacity of intestinal bacteria to produce diverse enzymes, a significant gap remains between these predictions and experimentally validated enzymatic activity. This disconnection underscores the need for systematic exploration of microbial ecosystems, supported by technological advancements and interdisciplinary collaboration. 

To address this gap, our study combined in silico and in vitro approaches to analyze the enzymatic potential of gut microbiota isolates. Genomic annotation data sourced from the National Center for Biotechnology Information (NCBI) database were analyzed to predict potential enzymatic activities of previously selected microbial cultivable species, providing a computational overview of their biochemical capabilities. These predictions were experimentally validated through targeted in vitro assays, focusing on seven specific enzymatic activities.

## 2. Materials and Methods

### 2.1. In Silico Whole Genome Sequences (WGS) Enzyme Searching Approach

A collection of cultured microorganisms from human gut microbiota was generated from fecal samples from children between 6 and 12 years old including those with normal-weight and obesity and with special tolerability to plasticizer xenobiotics [15]. They were growth in different culture media, including Brain−Heart Infusion (BHI), Man Rogosa and Sharpe (MRS), Reinforced Clostridial Medium (RCM), Gifu Anaerobic Medium modified with agar (GAMa), and Gifu Anaerobic Medium modified with gellan gum (GAMg) [16]. This procedure, based on the methodology carried out by López-Moreno et al. (2022) [17], enabled the strain isolation of BPA-tolerant colonies, which were taxonomically characterized using MALDI-TOF techniques and 16S rRNA complete gene sequencing. The resulting gut microbiota collection comprised all the isolates representing viable and long-term cultivable strains.

Genomic screening was performed using the species identified in this catalog as references species. Subsequently, for each cultured species, genomic records were sought in NCBI database, prioritizing those with complete chromosomes and type strains, as well as assemblies with the highest level of completeness available (≥98%). When type strain annotations were unavailable, the most complete assemblies were selected to ensure reliable genomic representation.

Genera were assessed for inclusion based on the availability of genomic annotations and sufficient species representation. Genera lacking genomic records in NCBI database at the time of analysis were excluded, as their absence limited the ability to properly evaluate enzymatic potential. Genera with less than three species were not included due to their low representation, deemed insufficient for reliable comparative analysis. This approach ensured the selection of genera with validate genomic data, facilitating accurate and meaningful genomic screening.

Seven major genera from microbiota were selected: *Bacillus*, *Bacteroides*, *Clostridium*, *Enterobacter*, *Enterococcus*, *Microbacterium*, and *Staphylococcus*. The selection was performed according to higher prevalence among isolates, probability of cultivation, known or hypothetical biotechnological applications, and relevant role in the context of the human gut microbiota. For the genus *Clostridium*, both “chromosome, complete genome” annotations and “whole genome shotgun sequences” were considered.

The enzyme screening strategy was conducted using a custom Microsoft PowerShell script designed to search for specific enzyme names within the annotation files. For this purpose, a specific directory was first created to store all downloaded genomic annotation files from the NCBI database, in GenBank format (.gb files). These files corresponded to selected strains, prioritizing type strains when available, or otherwise the strains with the highest-quality genome assemblies. Each annotation file contained detailed genomic information, including annotated genes, proteins, and enzyme references.

To streamline the search process, the script executed targeted, case-sensitive queries across the entire annotation dataset, allowing for the rapid identification of specific enzyme names within the genomic files. The following command was used:

PS C:\Users\Usuario> Get-ChildItem -Path [Directory path] -Recurse | Select-String -Pattern [Enzyme name] -CaseSensitive

where PS C:\Users\Usuario> corresponds to the PowerShell console prompt, indicating the current working directory on the local computer (in this case, “Usuario” refers to the username in a Spanish-language system), [Directory path] refers to the folder containing the genomic annotation files (.gb format) downloaded from NCBI, and [Enzyme name] corresponds to the target enzyme or protein to be searched within the annotation files. This process allowed the identification of matches between the genome annotations and the precompiled list of enzymes. Positive matches (+) and negative matches (-) were manually registered in an Excel checklist designed for comparative analysis.

This checklist included a comprehensive list of 364 biotechnologically relevant enzymes, along with their corresponding Enzyme Commission (EC) numbers, grouped by enzyme category (e.g., amylases, cellulases, esterases-lipases, nucleases, proteases, and xylanases). Each genome was cross-referenced against this list, recording the presence (+) or absence (-) of each enzyme. A subset of these enzymes is presented in Table 1, and the complete dataset is provided in Supplementary Materials.

**Figure 1:**
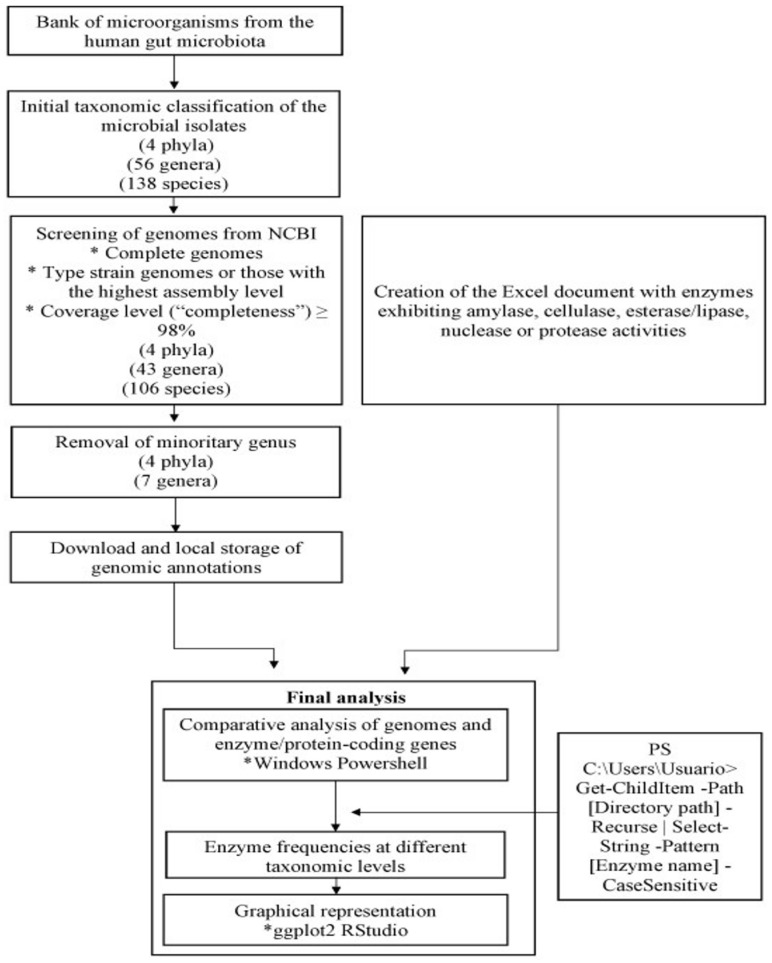
In silico workflow for the WGS enzyme searching analyses. *: Main criteria used for WGS selection and informatic tools employed during analysis.

The frequency of enzymes and proteins was graphically represented using the ggplot2 package in RStudio v. 4.3.2 [18]. The full workflow, from the taxonomic classification of species to the final graphical analysis of enzyme results, is illustrated in Figure 1.

### 2.2. In Vitro Culturing and Enzyme Activities

The enzymatic activities of amylase, cellulase, inulinase, lipase/esterase, DNase, protease, and gelatinase were evaluated for 93 isolates from the human gut microbiota collection.

Prior to the final enzymatic assays, a preliminary screening without OD standard-ization was conducted to qualitatively identify isolates displaying positive or negative enzymatic activities under differential media conditions. Based on these initial observations, for each differential medium tested in the final standardized assays, at least one isolate previously identified as positive and one as negative was included on the same plate. This approach served as an internal control system to validate the reliability of the enzymatic detection under the established conditions.

The isolates were cultured on Tryptic Soy Agar (TSA) at 37 °C for 24 h. The cells were then washed with a 0.9% saline solution and centrifuged three times at 14,000 rpm for 15 min. The cell concentration was adjusted to an optical density of OD600 nm = 0.25, and 10 µL aliquots were spotted onto the surface of differential media to test specific enzymatic activities. The relative enzymatic activity (REA) was calculated as the diameter of the clearing zone divided by the diameter of the microbial colony, measured in millimeters. All assays were performed in triplicate under strictly standardized conditions. The same isolates were spotted in identical order and position across the plates, ensuring uniform exposure to media, incubation time, and temperature. This rigorous approach was adopted to guarantee reproducibility, minimize technical variation, and provide robust comparative analyses. The results were categorized as excellent activity (REA > 5), good activity (REA > 2.0–5.0), or poor activity (REA < 2.0) [19].

**Figure 2:**
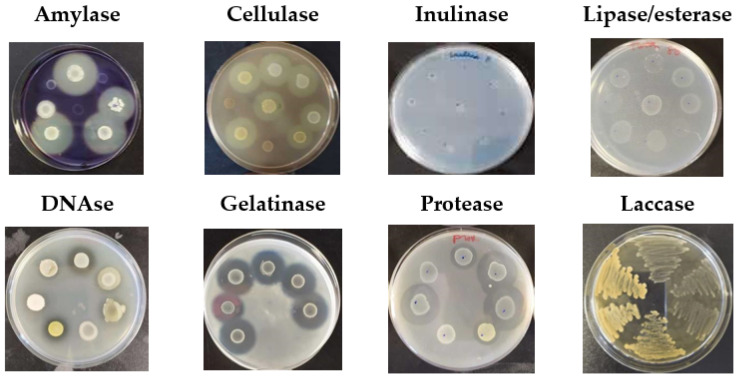
Phenotypic interpretation of enzymatic activities.

Amylase activity was evaluated according to Menasria et al. (2018) [20], with the following medium composition (g/L): soluble starch, 2; peptone, 5; yeast extract, 1; agar, 20. Colonies were incubated at 37 °C for 48 h and revealed using an iodine–potassium iodide solution (Lugol’s solution) [21]. Clear halos in the medium indicated positive amylase activity (Figure 2).

Cellulase activity was detected using a medium containing the following (g/L): carboxymethylcellulose, 5; NaNO_3_, 1; K_2_HPO_4_, 2; yeast extract, 0.5; glucose, 1; agar, 20 [20]. Colonies were incubated at 37 °C for 48 h and revealed with an iodine-potassium iodide solution. Clear halos around the colonies indicated positive cellulase activity [22] (Figure 2).

Inulinase activity was determined using a medium composed of the following (g/L): inulin, 2; (NH_4_)_2_SO_4_, 0.5; KH_2_PO_4_, 3; agar, 20 [20]. Colonies incubated at 37 °C for 48 h demonstrated inulinase capacity through visible growth on the medium [23] (Figure 2).

Lipolytic/esterase activity was tested using a medium containing the following (g/L): Tween 80 (0.1%), yeast extract, 1; agar, 20 [20]. Colonies incubated at 37 °C for 48 h formed turbid halos, indicating enzymatic activity [24] (Figure 2).

DNase activity was assessed using DNase agar (Thermo Scientific, Oxoid, Basingstoke, Hampshire, UK). Colonies incubated at 37 °C for 48 h were revealed with 1N HCl, producing clear halos around DNase-positive colonies, while the rest of the medium appeared opaque [25] (Figure 2).

Gelatinase activity was evaluated using a medium composed of the following (g/L): gelatin, 10; yeast extract, 1; agar, 20 [20]. Colonies incubated at 37 °C for 48 h formed clear halos after applying a 15% HgCl_2_ solution in 20% HCl [26] (Figure 2).

Protease activity was determined using a medium containing the following (g/L): skim milk, 10; yeast extract, 1; agar, 20 [20]. Colonies incubated at 37 °C for 24 h dis-played clear halos, indicating proteolytic activity [27].

Laccase production was qualitatively assessed on agar plates using Luria–Bertani Agar (LB) as the standard substrate for detecting this enzymatic activity. The plates contained 10 g/L tryptone, 10 g/L NaCl, 5 g/L yeast extract, 15 g/L agar, 0.01% guaiacol, and 0.35 mM CuSO_4_ [28]. After 7 days of incubation at 37 °C, brown-yellow coloration, indicating guaiacol oxidation, was monitored to confirm positive laccase activity, and negative isolates showed white coloration (Figure 2).

## 3. Results

### 3.1. In Silico WGS Enzyme Activity in Microbiota

#### 3.1.1. Amylases

**Figure 3:**
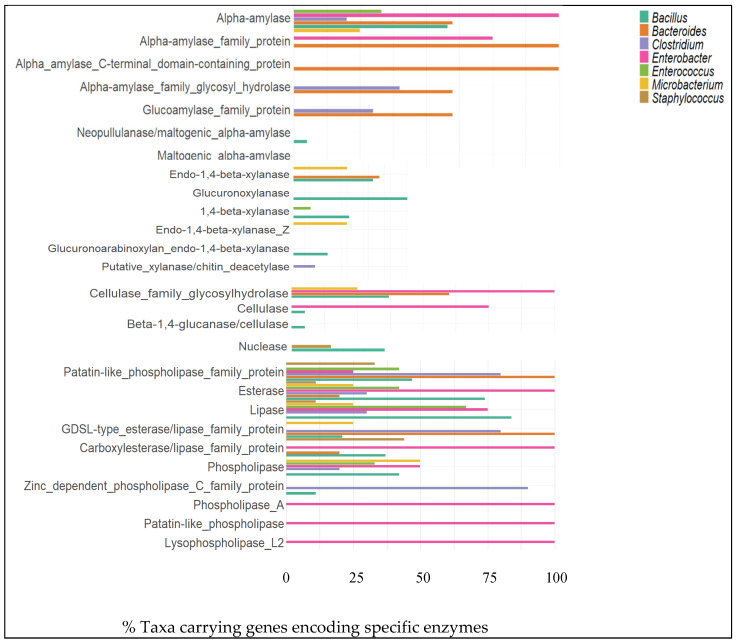
Enzymes related to amylase, xylanase, cellulose, nuclease, lipase activities and their distribution in percentages among the phyla Actinomycetota (n = 10), Bacillota (n = 67), Bacteroidota (n = 9), and Pseudomonadota (n = 15) and genera *Bacillus* (n = 19), *Bacteroides* (n = 5), *Clostridium* (n = 10), *Enterobacter* (n = 4), *Enterococcus* (n = 12), and *Microbacterium* (n = 4). n = number of taxa/WGSs.

Alpha-amylase and related proteins were present in microorganisms across all analyzed phyla. Alpha-amylase was predominant in the phylum *Pseudomonadota* (73%), followed by *Bacteroidota* (67%). Regarding the alpha-amylase C-terminal domain-containing protein, alpha-amylase family glycosyl hydrolase, and glucoamylase family protein, a high prevalence was observed in microorganisms belonging to the phylum *Bacteroidota*. Alpha-amylase appeared in most genera, reflecting its widespread presence. Notably, this enzyme was identified across all annotations of the genus *Enterobacter*. Similarly, 100% of microorganisms within *Bacteroides* contained both the alpha-amylase family protein and the alpha-amylase C-terminal domain-containing protein (Figure 3).

The amylase activity was detected in microbiota isolates belonging to the genera *Bacillus*, *Enterococcus*, *Escherichia*, *Staphylococcus*, *Masillia*, *Siminovitchia*, *Burkholderia*, and *Roultella*. Among the isolates, 34 exhibited good extracellular amylase activity, 2 showed poor activity, and 57 had no detectable amylase activity. A *Bacillus mojavensis* isolate exhibited strong amylase activity, with a REA value of 3.8, the highest observed for this activity. However, microbial isolates identified as *Burkholderia contaminans* and *Enterococcus durans* also showed good amylase activity, with REA values of 3.7 and 3.5, respectively. No microorganisms were found to demonstrate excellent amylase production (REA > 5.0) (Supplementary Materials).

#### 3.1.2. Xylanases

The analysis identified multiple xylan-degrading enzymes encoded in the genomes of various microorganisms associated with the human gut microbiota. Microorganisms related to the phylum Bacteroidota demonstrated the highest prevalence of endo-1,4-beta-xylanase, which was identified in 33% of genomic annotations, making it the enzyme with the highest prevalence. Other phyla, such as Bacillota and Actinomycetota, exhibited significant enzymatic potential. In Bacillota, glucuronoxylase was particularly abundant, observed in 15% of microbial annotations, while in Actinomycetota, both endo-1,4-beta-xylanase and endo-1,4-beta-xylanase Z were identified in 10% of genomic annotations. Among the xylan-degrading enzymes analyzed, endo-1,4-beta-xylanase was the most widely distributed, found in 40% of microorganisms related to genus *Bacteroides* and 37% of those belonging to the genus *Bacillus*. Additionally, glucuronoxylanase showed the highest overall representation, identified in 53% of genomic annotations, with notable abundance among microorganisms in the genus *Bacillus* (Figure 3).

#### 3.1.3. Cellulases

Cellulase-related enzymes were analyzed across several microbial phyla. Notably, Pseudomonadota and Bacteroidota exhibited the highest prevalence of cellulase family glycosylhydrolase, with more than 40% of taxa carrying this enzyme within the genomic annotations. The cellulase family glycosylhydrolase enzyme was identified in all genomic annotations related to microorganisms of the genus *Enterobacter*. Furthermore, 75% of these microorganisms exhibited a high prevalence of cellulase (Figure 3).

The cellulase activity was detected in microbiota isolates belonging to the genera *Bacillus*, *Enterococcus*, *Escherichia*, *Staphylococcus*, *Microbacterium*, *Lacticaseibacillus*, *Masillia*, *Siminovitchia*, *Micrococcus*, *Burkholderia*, and *Raoultella*. A total of 29 isolates exhibited good cellulase activity, 12 showed poor activity, and 52 had no detectable cellulase activity. Examining cellulase production, *Enterococcus avium* exhibited the highest activity, with a REA value of 3.3. Other isolates, such as *Bacillus velezensis* and *Enterococcus gilvus*, showed similar cellulase activity, with REA values of 3.1. No iso-lates demonstrated cellulase production rated as excellent (Supplementary Materials).

Inulinases: In the evaluation of inulinase activity, microbial growth, indicative of positive inulinase activity, was observed in 64 microbiota isolates. These isolates were identified as belonging to the genera *Bacillus*, *Enterococcus*, *Escherichia*, *Staphylococcus*, *Microbacterium*, *Lacticaseibacillus*, *Enterobacter*, *Masillia*, *Siminovitchia*, *Pseudomonas*, *Mi-crococcus*, *Burkholderia*, and *Raoultella* (Supplementary Materials).

#### 3.1.4. Nucleases

It was observed that all microorganisms analyzed in silico exhibited a wide diversity of nucleases, enzymes involved in the degradation of DNA, RNA, or other functions associated with these molecules. Nucleases were identified across all analyzed microbial phyla, with notable representation in 20% of microorganisms associated with the phylum Actinomycetota. At the genus level, nucleases were predominantly identified in 26% of microorganisms from the genus *Bacillus* and less frequently in 11% of microorganisms from the genus *Staphylococcus*. (Figure 3). 

The in vitro degradation of DNA was observed in the genera *Bacillus*, *Enterococcus*, *Escherichia*, *Staphylococcus, Siminovitchia*, *Microbacterium*, *Pseudomonas,* and *Raoultella*. However, good-DNA-degrading activity was limited to 6 isolates, while the remaining 32 exhibited poor or no activity (Supplementary Materials).

#### 3.1.5. Esterase/Lipase

Several enzymes, including esterases, patatin-like phospholipase family protein, GDSL-type esterase/lipase family protein, phospholipase, and carboxylesterase/lipase family protein, were identified in the phyla Actinomycetota, Bacillota, Bacteroidota, and Pseudomonadota. The distribution of these enzymes varies among the phyla, with Pseudomonadota and Bacteroidota exhibiting the highest levels of enzymes such as es-terase, phospholipase A, carboxylesterase/lipase family protein, phospholipase D fami-ly protein, lysophospholipase L2, patatin-like phospholipase, and patatin-like phos-pholipase family protein, GDSL-type esterase/lipase family protein, and phosphatidyl-inositol-specific phospholipase C, respectively. As shown in Figure 3, the distribution of enzymes across the evaluated genera demonstrates that esterases were present in genomic annotations of all seven genera. Notably, the genus *Enterobacter* exhibited a broader enzymatic repertoire, with all microorganisms within this group showing annotations for carboxylesterase/lipase family proteins, phospholipase A, patatin-like phospholipase, and lysophospholipase L2. *Microbacterium paraoxydans* showed the highest relative enzymatic activity (REA = 2.0), followed by *Raoultella ornithinolytica*. Of the 93 isolates tested, 49 showed poor enzymatic activity (REA < 2.0), while 43 showed no enzymatic activity. Most identified genera, such as *Bacillus*, *Escherichia*, *Enterococcus*, *Lacticaseibacillus*, *Enterobacter*, *Siminovitchia*, *Microbacterium*, *Micrococcus*, *Burkholderia*, and *Raoultella*, displayed poor enzymatic activity (Supplementary Materials).

#### 3.1.6. Proteases

The analysis of proteolytic enzymes and proteins identified a total of 279 components at the phylum level and 221 at the genus level across the 292 studied.

These were categorized into metalloproteases, cysteine proteases, serine proteases, and other enzymes/proteins. A detailed breakdown of their distributions across taxonomic levels is presented in the following sections.

##### Metalloproteases

In the analysis of metalloproteases, several noteworthy cases were identified based on their distributions across the four phyla and seven genera studied. Among these, the ATP-dependent zinc metalloprotease FtsH, the CPBP family intermembrane metalloprotease, and the SprT family zinc-dependent metalloprotease were particularly remarkable, as they were consistently present in microorganisms from all four analyzed phyla. In particular, enzymes such as ATP-dependent zinc metalloprotease FtsH and RIP metalloprotease RseP, exhibited notably high representation in microorganisms belonging to phyla Bacteroidota.

**Figure 4:**
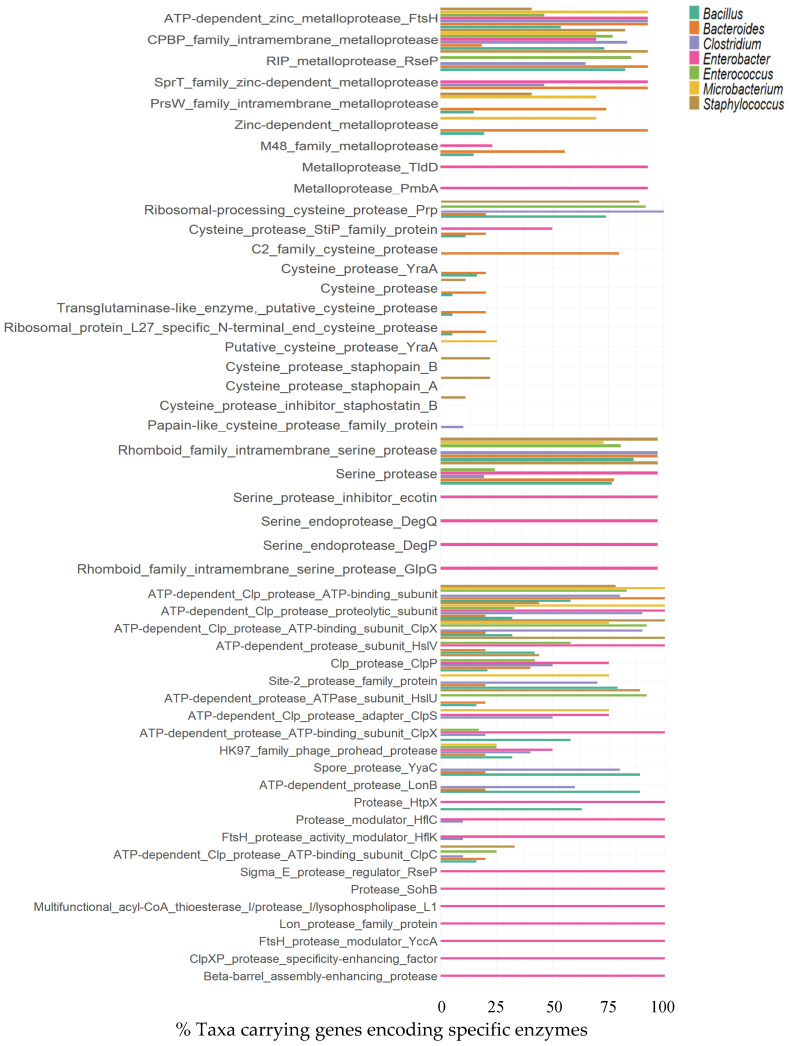
Main metalloprotease enzymes and their distribution in percentages analyzed across the phyla Actinomycetota (n = 10), Bacillota (n = 67), Bacteroidota (n = 9), and Pseudomonadota (n = 15) and genera *Bacillus* (n = 19), *Bacteroides* (n = 5), *Clostridium* (n = 10), *Enterobacter* (n = 4), *Enterococcus* (n = 12), *Microbacterium* (n = 4), and *Staphylococcus* (n = 9).

The metalloproteases FtsH and CPBP were identified in microorganisms from all seven evaluated genera. The FtsH enzyme was theoretically present in microorganisms belonging to the genera *Bacteroides* (100%), *Clostridium* (100%), *Enterobacter* (100%), *Microbacterium* (100%), *Bacillus* (58%), *Enterococcus* (50%), and *Staphylococcus* (44%). In contrast, the CPBP family intramembrane metalloprotease was not found across all analyzed annotations of these genera but showed notable prevalence in *Clostridium* (90%), *Staphylococcus* (89%), *Enterococcus* (83%), *Bacillus* (79%), *Enterobacter* (75%), *Microbacterium* (75%), and *Bacteroides* (20%) (Figure 4).

##### Cysteine Proteases

Cysteine proteases revealed a diverse distribution across microbial phyla. The most notable findings include the prevalence of the ribosomal processing cysteine protease Prp, detected in 82% of microorganisms from the phylum Bacillota, and the C2 family cysteine protease, found in 44% of microorganisms within phylum Bacteroidota. Other notable observations include the exclusive presence of the putative cysteine protease YraA in 10% of microorganisms from the phylum Actinomycetota. In contrast, enzymes such as cysteine protease staphopain B, staphopain A, and the cysteine protease inhibitor staphostatin B were found in smaller proportions (1–3%) within Bacillota. Additionally, 7% of microorganisms in the phylum Pseudomonadota exhibited the YopT-type cysteine protease domain-containing protein. The distribution of cysteine proteases reveals distinct trends across genera. The ribosomal-processing cysteine protease Prp was the most prevalent, identified in all genomic annotations of *Clostridium*, 92% of *Enterococcus*, 89% of *Staphylococcus*, and 74% of *Bacillus*. Notable enzymes include the cysteine protease StiP family protein, predominantly found in *Enterococcus*, and the C2 family cysteine protease, with high representation in *Bacteroides*. The cysteine protease YraA and other papain-like enzymes, such as staphopain A and B, were present in lower proportions, primarily in *Staphylococcus* and *Bacillus* annotations (Figure 4).

##### Serine Proteases

The distribution of taxa encoding serine proteases was analyzed, and rhomboid family intramembrane serine proteases were found to be the most widely distributed across the analyzed phyla, with near-universal representation in members of Bacteroidota and Bacillota, accounting for over 90% of taxa within these groups. Similarly, serine protease was broadly encoded across all phyla, with slightly lower representation in Actinomycetota and higher distribution among microorganisms belonging to the phylum Pseudomonadota. The most prevalent enzymes, such as rhomboid family in-tramembrane serine protease and serine protease, were broadly distributed across most taxa. Rhomboid family intramembrane serine protease was identified in all mi-croorganisms belonging to the genera Bacteroides, Clostridium, and Staphylococcus. Similarly, serine protease was present in all genomic annotations associated with *Enterobacter*. Other enzymes, including serine protease inhibitor ecotin, serine endoproteases DegQ and DegP, and rhomboid family intramembrane serine protease GlpG, were also detected in all Enterobacter annotations (Figure 4).

##### Other Proteases

The analysis of proteases distinct from metalloproteases, cysteine proteases, and serine proteases revealed a diverse array of enzymes and proteins with proteolytic activity. Among these, the ATP-dependent Clp protease ATP-binding subunit, ATP-dependent Clp protease ATP-binding subunit ClpX, ATP-dependent Clp protease proteolytic subunit, HK97 family phage prohead protease, and site-2 protease family protein showed different distributions among all four analyzed phyla.

In Bacillota, protease diversity was broader, with the ATP-dependent Clp protease ATP-binding subunit being the most prevalent (75%), followed by ClpX (64%) and ClpC (61%). Unique proteases, such as collagenase-like types and those linked to sporulation or stress response, were present but less common. In Bacteroidota, ATP-dependent proteases dominated, with the ATP-binding subunit and ClpX subunit being the most frequent (100% and 89%, respectively). The data reveal a variety of protease-related proteins across different bacterial species, with specific frequency distributions. For instance, only the ATP-dependent Clp protease proteolytic subunit showed distinct distribution patterns among all seven genera studied. Other protease-related proteins identified in all genomic annotations of the genus Enterobacter include the ATP-dependent Clp protease proteolytic subunit, ATP-dependent protease ATP-binding subunit ClpX, ClpXP protease specificity-enhancing factor, protease HtpX, protease SohB, ATP-dependent protease subunit HslV, protease modulator HflC, FtsH protease activity modulator HflK, sigma E protease regulator RseP, beta-barrel assembly enhancing protease, FtsH protease modulator YccA, Lon protease family protein, and multifunctional acyl-CoA thioesterase I/protease I/lysophospholipase L1 (Figure 4). Protease activity was detected across multiple genera, including *Bacillus*, *Escherichia*, *Enterococcus*, *Staphylococcus*, *Siminovitchia*, *Massilia*, *Microbacterium*, *Micrococcus*, *Burkholderia*, and *Raoultella.* The highest activity (REA up to 2.5) was observed in isolates such as *Bacillus thuringiensis* and *Escherichia coli*, while lower activity was noted in isolates like *Microbacterium paraoxydans* and *Micrococcus luteus*. Of the 93 isolates tested, 21 exhibited good protease activity, 19 showed poor activity, and 52 displayed no protease activity (Supplementary Materials).

Gelatinases: Gelatin degradation was observed in genus *Bacillus*, *Escherichia*, *Enterococcus*, *Staphylococcus*, *Lacticaseibacillus*, *Siminovitchia*, *Massilia*, *Microbacterium*, and *Raoultella.* Among the 48 isolates with prominent enzymatic activity, *Escherichia coli* showed the highest REA values, reaching up to 4.8 ± 0.00, followed by *Burkholderia contaminans* and *Massilia haematophila* (Supplementary Materials).

#### 3.1.7. Laccases

The analysis of multicopper oxidases revealed distinct patterns of enzyme distribution across microbial taxa. The phylum *Bacillota* exhibited the highest representation of the identified enzymes, with notable diversity across categories. Among the analyzed enzymes, multicopper oxidase (laccase) was predominantly associated with microorganisms from the phylum *Actinomycetota*, which it was detected in 20% of the taxa. In contrast, 7% of microorganisms from the phylum *Pseudomonadota* encoded genes for this enzyme. Notably, laccase was also the most prevalent enzyme within the phylum *Bacillota*, identified in 9% of genomic annotations.

Laccase was most frequently identified in taxa from the genus *Bacillus*, representing 21% of the analyzed annotations. Additionally, the outer spore coat copper-dependent laccase CotA was exclusively associated with members of *Bacillus*, highlighting its specialized role in spore coat formation and stress resilience.

**Figure 5:**
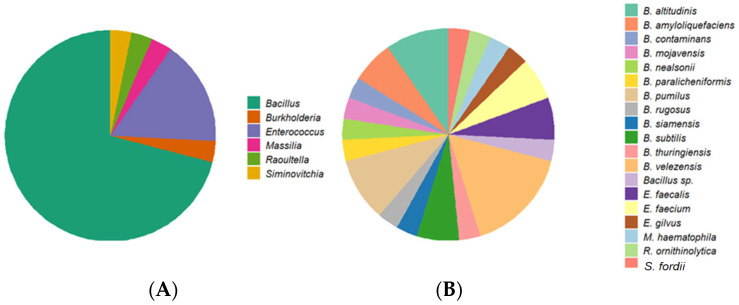
Distribution of laccase-producing isolates at the genus level (**A**). Distribution of laccase-producing isolates at the species level (**B**).

In contrast, genera such as *Clostridium* and *Staphylococcus* displayed relatively lower but consistent levels of laccase distribution (Figure 5).

Microorganisms exhibiting extracellular laccase activity were primarily associated with the phylum *Bacillota* (28 isolates), followed by *Pseudomonadota* (3 isolates). The genus *Bacillus* dominated, with 22 isolates identified, including *Bacillus altitudinis*, *Bacillus amyloliquefaciens*, *Bacillus mojavensis*, *Bacillus nealsonii*, *Bacillus paralicheniformis*, *Bacillus pumilus*, *Bacillus rugosus*, *Bacillus siamensis*, *Bacillus* sp., *Bacillus subtilis*, *Bacillus thuringiensis*, and prominently, *Bacillus velezensis*. Remarkable laccase producers were also observed in the genus *Enterococcus*, with isolates such as *Enterococcus faecalis*, *Enterococcus faecium*, and *Enterococcus gilvus*. Furthermore, other genera with extracellular laccase activity included *Siminovitchia fordii*, *Massilia haematophila*, *Burkholderia contaminans*, and *Raoultella ornithinolytica* (Figure 5).

### 3.2. In Vitro Enzyme Activity

**Figure 6:**
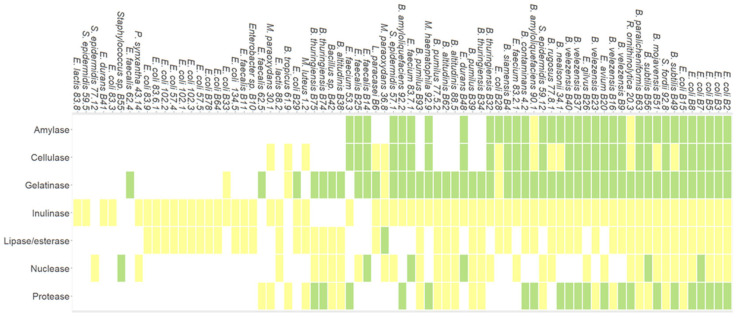
Heatmap of enzymatic activity from main strain isolates of human gut microbiota. Green: good activity REA > 2 < 4, Yellow: poor activity REA < 2; White: no activity REA = 0.

The screening of 93 human gut microbiota isolates for seven different hydrolytic activities revealed that 80.6% of the isolates were able to produce, at least one of the seven hydrolytic enzymes studied. Furthermore, 12.9% of the isolates showed the ability to degrade all substrates used to assess the seven enzymatic activities of interest. The 93 isolates were classified into three categories based on their enzymatic activities: high, low, or no detectable activity. A total of 34 isolates exhibited high amylase activity, 29 showed high cellulase activity, 48 displayed strong gelatinase activity, and 21 demonstrated high protease activity. For lipase/esterase, 50 isolates exhibited low activity, with no cases of high activity. Regarding nuclease activity, only six isolates showed high activity. Most of the remaining isolates displayed either low or no detectable enzymatic activity across the assays (Figure 6).

## 4. Discussion

Microbiota species comprise about 500–1000 different species [29] with specific enzymes and metabolic pathways, whose functionalities interact with host for symbiotic purposes [30]. Microorganisms within the gut microbiota play crucial roles at nutritional and physiological levels [31], enhancing nutrient bioavailability [32], supporting immune modulation, and maintaining gut homeostasis [2]. Their enzymatic diversity underpins these functions, enabling them to interact with complex dietary components. This diversity also extends to their broader ecological applications, including their capacity to produce high-value nutritional compounds and contribute to sustainable food systems through metabolic engineering and synthetic biology [33].

Among these microorganisms, members of the phyla Bacillota and Bacteroidota stand out as primary degraders of polysaccharides. Their genetic enrichment in carbohydrate-active enzyme (CAZyme) encoding genes, that enables them to metabolize a broad range of polysaccharides effectively [3].

Among these, the genus Bacillus is particularly prominent for starch degradation. Experimental enzymatic activity assays confirmed that isolates with the highest amylase activity belonged to the genus *Bacillus*, with *B. mojavensis* exhibiting the greatest capacity. These findings align with prior studies, which reported relative enzymatic activity (REA) values ranging from 1.0 to 6.3 in starch-enriched media [19,34,35]. The consistency across studies underscores the pivotal role of *Bacillus* species as efficient amylase producers with potential industrial relevance in food processing.

Theoretical analyses identified microorganisms from the phyla Bacillota and Pseudomonadota, specifically the genera *Bacillus* and *Enterobacter*, as potential cellulase producers. Experimental results confirmed *Bacillus* isolates as effective cellulose degraders; however, interestingly, *Enterococcus* isolates (*E. avium* B20 and *E. gilvus* B26) exhibited equal or superior cellulase activity. While *Enterococcus* has been previously noted for cellulase production, its prominent activity in this study highlights an under-explored enzymatic potential. This finding suggests that members of *Enterococcus*, traditionally associated with non-cellulolytic roles, may contribute more to cellulose degradation than previously recognized [36]. Future studies should also evaluate the broader range of cellulase-related enzymes within the gut microbiota. Expanding the search to encompass other glycosyl hydrolases and cellulase families could uncover complementary or synergistic enzymatic activities contributing to cellulose degradation.

All taxa analyzed displayed a broad repertoire of enzymes involved in the degra-dation of nucleic acids (DNA and RNA) and related processes. However, the specific enzyme “nuclease” was not widely distributed among the taxa examined, primarily re-stricted to members of the phylum Actinomycetota and microorganisms of the genus *Bacillus*. Experimentally, *Bacillus* isolates exhibited the highest DNA-degrading activi-ty, consistent with theoretical predictions. While these findings provide valuable insights, they also underscore an important limitation: focusing only on the enzyme “nuclease” may underestimate the overall nucleic acid degradation capacity of microbial taxa. Nucleases represent a diverse group of enzymes, and future studies should expand predictive efforts to include other enzymes within this family. This approach would provide a more comprehensive understanding of microbial potential for DNA and RNA degradation and might uncover previously overlooked patterns of enzymatic activity across taxa. For instance, other types of nucleases, such as endonucleases and exonucleases with varying specificities and mechanisms, could play significant roles in extracellular nucleic acid turnover. Broader screening for these enzymes could better elucidate the functional roles of different microorganisms within ecological and biogeochemical contexts [37,38]. Expanding the predictive scope to include a wider array of nuclease-related enzymes could also improve the accuracy of experimental validation and theoretical models. This would ultimately refine our understanding of how microbial communities contribute to nucleic acid cycling and open new avenues for exploring enzymatic diversity with potential biotechnological applications [39].

Notable discrepancies emerged between theoretical predictions and observed enzymatic activities for lipid and gelatin degradation.

For lipid degradation, theoretical genome analyses identified a diverse repertoire of lipid-degrading enzymes, including phospholipases, lipases, esterases, and mono-acylglycerol and triacylglycerol-lipases. However, experimental assays using Tween 80 as the substrate revealed significantly reduced lipolytic activity among the isolates. Several factors may explain this inconsistency.

First, enzyme activation conditions such as specific cofactors, pH, temperature, or redox environments required for optimal enzyme function may not have been fully replicated in vitro [40]. Certain lipases, for example, require emulsified natural triglyc-erides and interfacial activation at the lipid–water interfaces, which Tween 80—a synthetic, highly soluble ester—does not adequately mimic [38]. Additionally, substrate specificity varies widely even among annotated lipase/esterase enzymes; some show high selectivity for long-chain fatty acids or structured lipids that are absent in the testing medium [39].

Second, annotation errors and functional mispredictions can occur in genomic analyses. Many bioinformatic pipelines assign lipase/esterase annotations based solely on conserved motifs (such as the GXSXG serine hydrolase motif), which are also pre-sent in non-lipolytic enzymes, leading to potential overprediction [41,42,43]. Thus, experimental verification remains essential to validate putative lipolytic functions.

To bridge these gaps, future studies should diversify the lipid substrates tested (e.g., natural triglycerides and phospholipids) and modify assay conditions to better simulate physiological environments, including interfacial emulsions, bile salts, and cofactor supplementation. Additionally, transcriptomic or proteomic analyses could be employed to confirm the actual expression and functionality of the predicted lipid-degrading enzymes under the experimental conditions.

In contrast, gelatin degradation displayed an unexpected trend: numerous isolates exhibited strong gelatinase activity, even though genome-based predictions failed to anticipate such a function.

Several factors could account for this discrepancy. Unannotated genes or novel proteolytic enzymes not captured by standard databases may mediate gelatin degradation. Certain generalist proteases, while not classified specifically as gelatinases, can hydrolyze gelatin due to its unfolded, denatured collagen structure. Moreover, horizontal gene transfer of protease genes from environmental or gut sources could con-tribute to unpredicted gelatinolytic capabilities [44].

This highlights a critical limitation of in silico functional annotation: reliance on known sequences restricts the discovery of novel enzymatic activities. Consequently, our findings emphasize the need for experimental assays to complement bioinformatic predictions and advocate for expanded genomic mining approaches, such as de novo functional prediction methods and machine learning tools capable of identifying non-canonical enzyme families.

Proteolytic enzyme analysis identified a wide range of microorganisms involved in producing metalloproteases, cysteine proteases, and serine proteases, among others [45]. Experimental results confirmed that protease activity was distributed across various genera, including *Bacillus*, *Escherichia*, *Enterococcus*, *Staphylococcus*, *Siminovitchia*, *Massilia*, *Microbacterium*, *Micrococcus*, *Burkholderia*, and *Raoultella*, aligning with theoretical expectations. 

This diversity reflects the broad enzymatic capabilities of gut microbiota, con-sistent with findings from Li et al. (2024) [46], which demonstrated the phylogenetic diversity of microorganisms involved in amino acid metabolism. The overlap between protease-producing and amino acid-metabolizing genera, such as *Bacillus* and *Entero-coccus*, highlights a shared ecological strategy for accessing protein-derived nutrients in the gut environment. While protease activity has significant biotechnological potential, particularly in optimizing industrial processes, it is essential to consider its implications for human health. Proteases play a key role in protein hydrolysis, liberating free amino acids that may contribute to shifts in host amino acid homeostasis. Recent studies, including that by Li et al. (2024) [46], suggest that gut microbial metabolism of amino acids, influenced by enzymes such as proteases, can affect host physiology, including glucose tolerance and nutrient availability. Such interactions underscore the dual nature of protease activity: offering industrial and therapeutic opportunities while potentially contributing to physiological disorders in humans [47,48].

This emphasizes the need for balanced applications that weigh scientific and technical benefits against health considerations, including animal health improve-ments and food efficiency optimization [49]. Integrating insights from microbial genet-ics and metabolomics, as proposed by Li et al. [46], could aid in developing strategies to modulate protease activity, minimizing health risks while maximizing biotechnological benefits [49].

There is a useful platform that facilitates gut microbiota-specific enzyme discovery, biochemical activity annotations, and potential industrial or biopharmaceutical applications [49]. Interestingly, several authors described many specific enzymes, such as serine hydrolases, bile salt hydrolases, β-glucuronidases, and serine-type endopeptidases that were explored for the relationship between host health and target enzymes from the gut microbiome [50]. Discovering the microbial enzymes driving drug toxicity with activity-based protein profiling [51]. Globally, various microorganisms such as *Bacillus subtilis*, *Bacillus paralicheniformis*, *Bacillus mojavensis*, *Escherichia coli*, *Siminovitchia fordii*, and *Raoultella ornithinolytica* have demonstrated remarkable multi-enzyme activities. Notably, isolates producing amylase often co-express complementary enzymatic activities, including cellulase, inulinase, and gelatinase, highlighting their versatility in substrate degradation. These microorganisms are highly valued in the food industry for their ability to enhance the nutritional and functional properties of processed foods and animal feeds, and their multi-functional enzymatic profiles have been extensively documented in industrial microbiology and biotechnology [52].

Beyond enzymes involved in carbohydrate, lipid, and protein degradation, laccase emerges as a particularly versatile and valuable enzyme, owing to its broad substrate specificity and catalytic capabilities. Its ability to oxidize phenolic and non-phenolic compounds underpins its extensive industrial applications, including bioremediation, biosensors, and the transformation of complex chemical structures [53].

In this study, microorganisms of the genus *Bacillus* were identified as predominant producers of laccases within the analyzed fecal microbiota. This finding aligns with the expanding understanding of gut microbiota as a reservoir for biotechnologically significant enzymes. Historically, most studies and applications of laccases have focused on fungal sources, given their well-established role in industrial and environmental applications. However, bacterial laccases, particularly those derived from *Bacillus*, are rapidly gaining prominence [54,55]. Their unique biochemical properties, combined with the accessibility of genetic tools for bacterial systems, present a compelling alternative to traditional enzymatic sources.

The insights gained from this study highlight the gut microbiota as a potential reservoir of diverse enzymatic capabilities, including but not limited to laccase production, involved in specific complex polymer degradation, with high add value in several industries [56]. This enzymatic potential positions gut-derived *Bacillus* laccases and other enzymes as promising candidates for biotechnological innovation across various sectors [55,57]. However, translating this potential into practical applications requires addressing several challenges. These include the development of scalable and cost-effective methods to harness microbial enzymes, particularly laccases, and the transfer of this knowledge to industries such as food technology by engineering safe, genetically modified microorganisms with enhanced enzyme expression [58].

Integrating computational genomics with experimental enzyme validation, this study offers insights into the regulatory mechanisms of enzyme expression in gut-resilient microbes. It underscores the importance of bridging predictive and functional analyses to ensure the reliability of computational annotations.

## 5. Conclusions

The gut microorganisms from the four phyla analyzed in this study contained multi-enzyme activities, highlighting the production of amylases, which often co-express complementary enzymatic activities, including cellulases, inulinases, and gelatinases, demonstrating their versatility in substrate degradation.

Specific gut taxa showed enzymes involved in complex polymer degradation, such as laccases, which hold significant value in several industries.

Integrative approaches are essential for harnessing the enzymatic repertoire of gut microbiota, paving the way for targeted applications in medicine, agriculture, and food

## Figures and Tables

**Table 1:** Biotechnologically relevant enzymes and their EC numbers.

Enzyme Name	EC Number
Alpha-amylase	3.2.1.1
Alpha-amylase family glycosyl hydrolase	3.2.1.-
Alpha amylase C-terminal domain-containing protein	3.2.1.-
Neopullulanase/maltogenic alpha-amylase	3.2.1.135/3.2.1.133
Maltogenic alpha-amylase	3.2.1.133
Glucoamylase family protein	3.2.1.-
Alpha-amylase family protein	3.2.1.-
Alpha-amylase precursor	3.2.1.-
Putative glycanase or glycogenase with amylase domain	3.2.1.-
Maltohexaose-producing amylase	3.2.1.-
Family 15 glucoamylase	3.2.1.-
Alpha amylase, catalytic domain protein	3.2.1.-
Cellulase	3.2.1.4
Cellulase family glycosylhydrolase	3.2.1.-
Beta-1,4-glucanase/cellulase	3.2.1.4
Nuclease	3.1.-
Esterase	3.1.1.1
Lipase	3.1.1.3
Phospholipase C, phosphocholine-specific	3.1.4.3
Phospholipase	3.1.1.-
Phospholipase D family protein	3.1.4.4
Lipase chaperone	3.1.1.-
Patatin-like phospholipase family protein	3.1.1.3
Triacylglycerol lipase	3.1.1.3
GDSL-type esterase/lipase family protein	3.1.1.3
Lysophospholipase	3.1.1.5
Carboxylesterase/lipase family protein	3.1.1.1-
Phospholipase C	3.1.4.3
Serine protease, patatin-like phospholipase family protein	3.1.1.5
Lysophospholipase-like family protein, putative	3.1.2.22
Minor cardiolipin synthetase (phospholipase D family)	3.1.4.-
Conserved lipase family protein	3.1.1.3
Zinc-dependent phospholipase C family protein	3.1.4.3
Lipase family protein	3.1.1.3
Phospholipase D-like domain-containing protein	3.1.4.-
Spore germination lipase	3.1.1.-
Phosphatidylinositol-specific phospholipase C	3.1.4.11
Phospholipase/carboxylesterase	3.1.1.-
Phospholipase A2 family protein	3.1.1.4-

## Data Availability

The original contributions presented in this study are included in the article/Supplementary Material. Further inquiries can be directed to the corresponding author.

## Supplementary Materials

The following supporting information can be downloaded at: https://www.mdpi.com/article/10.3390/foods14101794/s1, Table S1: Complete list of analyzed enzymes and EC numbers; Table S2: Gut microbiota isolates with amylase activity; Table S3: Gut microbiota isolates with cellulase activity; Table S4: Gut microbiota isolates with inulinase activity; Table S5: Gut microbiota isolates with nuclease activity; Table S6: Gut microbiota isolates with lipolytic activity; Table S7: Gut microbiota isolates with proteolytic activity; Table S8: Gut microbiota isolates with gelatinase activity.
