# Supplementary material for: Analysis of Human Gut Microbiota Enzymes for Biotechnological and Food Industrial Applications
Source: Foods. 2025 May 18;14(10):1794. doi: 10.3390/foods14101794 (PMC12111023; doi:10.3390/foods14101794)
Supplement: Supplementary file 1 [file foods-14-01794-s001.zip › foods-3589484-supplementary.pdf]

Table S1. Complete list of analyzed enzymes and EC numbers.

Enzyme	EC number
Alpha-amylase	3.2.1.1
Alpha-amylase family glycosyl hydrolase	3.2.1.-
Alpha amylase C-terminal domain-containing protein	3.2.1.-
Neopullulanase / maltogenic alpha-amylase	3.2.1.135/3.2.1.133
Maltogenic alpha-amylase	3.2.1.133
Glucoamylase family protein	3.2.1.-
Alpha-amylase family protein	3.2.1.-
Alpha-amylase precursor	3.2.1.-
Putative glycanase or glycogenase with amylase domain	3.2.1.-
Maltohexaose-producing amylase	3.2.1.-
Family 15 glucoamylase	3.2.1.-
Alpha amylase, catalytic domain protein	3.2.1.-
Cellulase	3.2.1.4
Cellulase family glycosylhydrolase	3.2.1.-
Beta-1,4-glucanase/cellulase	3.2.1.4
Nuclease	3.1.-
Esterase	3.1.1.1
Lipase	3.1.1.3
Phospholipase C, phosphocholine-specific	3.1.4.3
Phospholipase	3.1.1.-
Phospholipase D family protein	-
Lipase chaperone	3.1.1.-
Patatin-like phospholipase family protein	-
Triacylglycerol lipase	3.1.1.3
GDSL-type esterase/lipase family protein	-
Lysophospholipase	3.1.1.5
Carboxylesterase/lipase family protein	-
Phospholipase C	3.1.4.3
Serine protease, patatin-like phospholipase family protein	-
Lysophospholipase-like family protein, putative	-
Minor cardiolipin synthetase (phospholipase D family)	3.1.4.-
Conserved lipase family protein	-
Zinc dependent phospholipase C family protein	-
Lipase family protein	-
Phospholipase D-like domain-containing protein	3.1.4.-
Spore germination lipase	3.1.1.-
Phosphatidylinositol-specific phospholipase C	3.1.4.11
Phospholipase/carboxylesterase	3.1.1.-
Phospholipase A2 family protein	-
GDSL family lipase	3.1.1.-
Lysophospholipase L2	3.1.1.5
Phospholipase A	3.1.1.4
Patatin-like phospholipase	3.1.1.-
Patatin family phospholipase	3.1.1.-
GDSL-like lipase/acylhydrolase	3.1.1.-
GDSL-like lipase/acylhydrolase family	3.1.1.-
Phospholipase D/transphosphatidylase	3.1.4.4
Phospholipase, patatin family	3.1.1.-
Putative lipase	-
Outer membrane phospholipase A	3.1.1.32
Phospholipase D	3.1.4.4

Esterase_lipase family protein	3.-.-.-
Esterase/lipase	3.1.1.1/3.1.1.3
Putative lysophospholipase	-
Monoacylglycerol lipase	3.1.1.23
Phospholipase C 3	3.1.4.-
Phosphatidylinositol-specific phospholipase C/glycerophosphodiester phosphodiesterase family protein	3.1.4.11/3.1.4.-
Lipase maturation factor family protein	-
Phospholipase D domain protein	3.1.4.-
Putative thermostable monoacylglycerol lipase	-
YSIRK domain-containing triacylglycerol lipase	3.1.1.-
Inulinase	3.2.1.7
Gelatinase	3.4.24.-
Pectinase	3.2.1.15
Protease	3.4.-.-
ATP-dependent protease	3.4.-.-
ATP-dependent Clp protease proteolytic subunit	3.4.21.92
ATP-dependent protease ATP-binding subunit ClpX	3.4.-.-
Tail-specific protease	3.4.21.102
ClpXP protease specificity-enhancing factor	3.4.-.-
Protease HtpX	3.4.-.-
Protease SohB	3.4.-.-
ATP-dependent Clp protease adapter ClpS	3.4.21.92
ATP-dependent Clp protease ATP-binding subunit	3.4.21.92
RIP metalloprotease RseP	3.4.24.-
Rhomboid family intramembrane serine protease	3.4.21.-
Protease Lon-related BREX system protein BrxL	3.4.-.-
HK97 family phage prohead protease	-
CPBP family intramembrane metalloprotease	-
ATP-dependent zinc metalloprotease FtsH	3.4.24.-
Clp protease ClpP	3.4.21.-
Retropepsin-like aspartic protease	3.4.23.-
Serine protease	3.4.21.-
ATP-dependent Clp protease ATP-binding subunit ClpC	3.4.-.-
Spore protease YyaC	3.4.-.-
Zn-dependent protease	3.4.-.-
Zinc metalloprotease HtpX	3.4.-.-
ATP-dependent protease LonB	3.4.-.-
ATP-dependent Clp protease ATP-binding subunit ClpX	3.4.-.-
PrsW family intramembrane metalloprotease	3.4.-.-
Site-2 protease family protein	-
Ribosomal-processing cysteine protease Prp	3.4.22.-
Glutamic-type intramembrane protease PrsW	3.4.-.-
XkdF-like putative serine protease domain-containing protein	-
Zinc-dependent metalloprotease	3.4.24.-
S1C family serine protease	3.4.21.-
ATP-dependent protease ATP-binding subunit ClpC	3.4.-.-
Cysteine protease StiP family protein	3.4.22.-
ATP-dependent protease subunit HslV	3.4.25.2
ClpP family protease	3.4.21.-
M48 family metalloprotease	3.4.24.-
Trypsin-like serine protease	3.4.21.-
Intramembrane metalloprotease PrsW	3.4.24.-

Protease adaptor protein SpxH	3.4.-.-
ATP-dependent protease ATP-binding subunit ClpE	3.4.-.-
M6 family metalloprotease domain-containing protein	3.4.24.-
ATP-dependent protease ATPase subunit HslU	3.4.-.-
ATP-dependent protease proteolytic subunit HslV	3.4.25.-
YhfC family intramembrane metalloprotease	3.4.24.-
Neutral protease NprB	3.4.24.-
RIP metalloprotease	3.4.24.-
Sporulation-specific protease PrtG	3.4.-.-
Protease synthase and sporulation negative regulatory protein PaiA	3.4.-.-
tRNA(NNU) t(6)A37 threonylcarbamoyladenosine modification, protease involved in TsaD function	3.4.-.-
Protease family protein	3.4.-.-
Transglutaminase-like enzyme, putative cysteine protease	-
Transcriptional regulator of extracellular protease production, sporulation and bacilysin production (MarR family)	3.4.-.-
Putative metalloprotease YhfN	-
Serine alkaline protease subtilisin E, subtilisin E	3.4.21.-
Cell wall-associated protease WprA	3.4.21.-
Zinc metalloproteinase aureolysin, extracellular neutral protease B	3.4.24.-
Membrane bound serine protease Do, quality control protease and chaperone (heat-shock protein)	3.4.-.-
Major intracellular serine protease precursor, IspA like	3.4.21.-
Membrane protease HtpX	3.4.24.-
ATP-dependent ClpE protease (class III stress response)	3.4.-.-
Bacillolysin, extracellular neutral metalloprotease	3.4.24.-
Serine protease, patatin-like phospholipase domain containing protein	3.4.-.-
Lon-like protease with PDZ domain	3.4.-.-
Two-component ATP-dependent protease (N-terminal serine protease)	3.4.-.-
Two-component ATP-dependent protease (ATPase and chaperone)	3.4.-.-
Inner membrane zinc metalloprotease required for the extracytoplasmic stress response mediated by sigma(W)	3.4.-.-
Putative zinc protease	-
Zinc protease	3.4.24.-
Putative zinc protease YmfH	-
Alkaline serine protease	3.4.21.-
SOS-response repressor and protease LexA	3.4.-.-
Carboxy-terminal processing protease CtpA	3.4.21.102
Protease required for RsiW anti-sigma(W) degradation	3.4.-.-
CAAX amino terminal protease family protein	-
Rhomboid family serine protease	3.4.21.-
Putative membrane-bound ClpP-class protease NfeDA	-
Spore germination protease	3.4.-.-
Putative protease large subunit YrrO	-
Putative protease small subunit YrrN	-
Ribosomal protein L27 specific N-terminal end cysteine protease	3.4.22.-
Class III heat-shock ATP-dependent LonA protease	3.4.-.-
Spore-specific ATP-dependent protease LonB	3.4.-.-
Protein unfolding ATPase required for presentation of proteins to proteases, Maxwell's demon	3.4.-.-
ATP-dependent Clp protease proteolytic subunit, Maxwell's demon	3.4.-.-

Protease IV, signal peptide peptidase	3.4.-.-
Putative membrane protease YugP	-
Protease-associated chaperone for the extracytoplasmic folding and quality control of exported proteins	3.4.-.-
PDZ-containing carboxyl-terminal protease processing protease (Zn(II))	3.4.-.-
Stage II sporulation protein related to metalloproteases (SpoIIQ)	3.4.-.-
Putative membrane zinc metalloprotease YwhC	-
Extracellular serine protease Vpr	3.4.21.-
Putative membrane protease YxkI	-
Sporulation membrane serine protease	3.4.21.-
ICEBs1 mobile element: site-specific protease cleaving ImmR	3.4.-.-
Putative integral inner membrane protease	-
Spore protease GPR related protein	3.4.-.-
Protease synthase/sporulation negative transcriptional regulator PaiB	3.4.-.-
SepM family pheromone-processing serine protease	3.4.21.-
Collagenase-like protease	3.4.-.-
Extracellular metalloprotease Mpr	3.4.24.-
Serine protease HtrA	3.4.21.-
Serine protease Isp	3.4.21.-
Neutral metalloprotease NprE	3.4.24.-
Protease DdcP	3.4.-.-
ATP-dependent protease subunit ClpQ	3.4.21.-
Serine protease AprX	3.4.21.-
Rhomboid protease YqgP	3.4.21.105
Cysteine protease YraA	3.4.22.-
Stage IV sporulation intramembrane metalloprotease SpoIVFB	3.4.24.-
Carboxy-terminal processing protease CtpB	3.4.21.102
Serine protease Vpr	3.4.21.-
Serine protease HtrC	3.4.21.-
CAAX protease	3.4.-.-
Zinc metalloprotease	3.4.24.-
Serine protease Do-like protein HtrB	3.4.21.-
Minor protease Epr	3.4.-.-
Zinc metalloprotease LiaK	3.4.24.-
Clp protease	3.4.21.92
CPBP family intramembrane metalloprotease domain-containing protein	3.4.-.-
Cysteine protease	3.4.22.-
Lon protease	3.4.21.53
Clp protease ATPase	3.4.-.-
Clp protease ClpX	3.4.-.-
S28 family serine protease	3.4.21.-
SprT family zinc-dependent metalloprotease	3.4.24.-
C2 family cysteine protease	3.4.22.-
Aspartyl protease family protein	-
Protease inhibitor I42 family protein	-
Tetratricopeptide repeat-containing serine protease family protein	3.4.21.-
Metalloprotease	3.4.24.-
Pepsin/retropepsin-like aspartic protease family protein	3.4.23.-
Metalloprotease family protein	3.4.24.-
DO serine protease	3.4.21.-

Protease or peptidase	3.4.-.-
Putative Zn-dependent protease	-
Putative metalloprotease	-
Putative ATP-dependent serine protease	-
ATP-dependent Clp protease proteolytic subunit 2	3.4.21.92
Protease II	3.4.-.-
Membrane metalloprotease yxkI	3.4.24.-
Matrixin family metalloprotease	3.4.24.-
M57 family metalloprotease	3.4.24.-
Membrane-associated protease 1	3.4.-.-
DUF2268 domain-containing putative Zn-dependent protease	-
Protease complex subunit PrcB family protein	3.4.-.-
Protease modulator HflC	3.4.-.-
FtsH protease activity modulator HflK	3.4.-.-
PDZ serine protease	3.4.21.-
Sigma E protease regulator RseP	3.4.24.-
Serine endoprotease DegP	3.4.21.-
Metalloprotease PmbA	3.4.-.-
DUF1471 family protease activator YjfN	3.4.-.-
Rhomboid family intramembrane serine protease GlpG	3.4.21.-
Metalloprotease TldD	3.4.24.-
Serine endoprotease DegQ	3.4.21.-
Beta-barrel assembly-enhancing protease	3.4.-.-
Serine protease inhibitor ecotin	3.4.21.-
ATP-dependent Clp protease ATP-binding subunit ClpA	3.4.-.-
FtsH protease modulator YccA	3.4.-.-
Lon protease family protein	-
Multifunctional acyl-CoA thioesterase I/protease I/lysophospholipase L1	3.4.-.-
Hydrogenase 1 maturation protease	3.4.-.-
ATP-dependent Clp protease adaptor protein ClpS	3.4.-.-
Predicted Zn-dependent protease (DUF2268)	3.4.-.-
Hydrogenase maturation protease superfamily	3.4.-.-
Membrane protein implicated in regulation of membrane protease activity	-
RIP metalloprotease RasP	3.4.24.-
ATP-dependent Zn protease	3.4.-.-
Rhomboid protease GluP	3.4.21.105
Neutral protease A, bacillolysin	3.4.-.-
Periplasmic trypsin-like serine protease	3.4.21.-
Subtilisin like protease	3.4.21.-
Peptidase M22, glycoprotease	3.4.24.-
Regulatory protease	-
Putative cell division protease FtsH	-
Clostripain family protease	3.4.-.-
Putative Zn-dependent protease-like protein	-
Lon protease 2	3.4.-.-
Sporulation protease LonB	3.4.-.-
ATP-dependent protease La	3.4.-.-
Carboxyl-terminal protease	3.4.21.102
Putative zinc-dependent protease	-
Caax amino protease family protein	-
YhfC family glutamic-type intramembrane protease	3.4.-.-

Hydrogenase maturation protease	3.4.-.-
Lon family ATP-dependent protease	3.4.-.-
Retroviral-like aspartic protease family	3.4.23.-
CAAX protease family protein	-
Zinc-dependent protease	-
Papain-like cysteine protease family protein	-
Caudovirus prohead protease	-
Phage protease	-
Zinc metalloprotease HtpX family protein	-
Omptin family outer membrane protease	3.4.-.-
Serine protease do-like htrA	3.4.21.-
ATP-dependent Clp protease ATP-binding subunit ClpE	3.4.-.-
PfpI family intracellular protease	3.4.-.-
Glycoprotease/Kae1 family metallohydrolase	3.4.24.-
CPBP family glutamic-type intramembrane protease	3.4.-.-
ATP-dependent protease ATP-binding subunit HslU	3.4.-.-
DJ-1/PfpI/YhbO family deglycase/protease	3.4.-.-
Spore protease-like protein	-
Bile acid germinant receptor pseudoprotease CspC	3.4.21.-
Bifunctional germination protease/germinant receptor pseudoprotease CspBA	-
PrsW family glutamic-type intramembrane protease	3.4.-.-
Serine endoprotease DegS	3.4.21.-
Protease FtsH-inhibitory lysogeny factor CIII	3.4.-.-
Omptin family outer membrane protease OmpT	3.4.-.-
Serine endoprotease (protease Do), membrane-associated	3.4.21.-
Protease involved in processing C-terminal end of HycE	3.4.-.-
DNA strand exchange and recombination protein with protease and nuclease activity	3.4.-.-
Putative protease, membrane anchored	-
Putative phage pro-head protease	-
ATPase and specificity subunit of ClpA-ClpP ATP-dependent serine protease, chaperone activity	3.4.21.-
Putative ATP-dependent protease	-
Carboxy-terminal protease for penicillin-binding protein 3	3.4.-.-
Ecotin, a serine protease inhibitor	-
DNA-binding ATP-dependent protease La	3.4.21.53
ATPase and specificity subunit of ClpX-ClpP ATP-dependent serine protease	3.4.21.-
Proteolytic subunit of ClpA-ClpP and ClpX-ClpP ATP-dependent serine proteases	3.4.21.-
Putative ATP-dependent Clp protease proteolytic subunit (Endopeptidase Clp) of prophage	-
Protease III	3.4.-.-
Putative membrane protease	-
Putative intracellular protease	-
Protease, ATP-dependent zinc-metallo	3.4.24.-
Serine endoprotease, periplasmic	3.4.21.-
Putative intramembrane serine protease	-
Putative protease involved in cellulose biosynthesis	-
Protease with a role in cell division	3.4.-.-
Peptidase component of the HslUV protease	3.4.-.-
Molecular chaperone and ATPase component of HslUV protease	3.4.-.-

Modulator for HflB protease specific for phage lambda cII repressor	-
Putative ATP-dependent Clp protease proteolytic subunit (Endo-peptidase Clp)	-
Proteasome-type protease	3.4.-.-
Putative serine protease	-
ATP-dependent protease ATP-binding subunit	3.4.-.-
ATP-dependent protease peptidase subunit	3.4.-.-
FtsH protease regulator HflK	-
FtsH protease regulator HflC	-
Serine endoprotease	3.4.21.-
Putative protease	-
Putative intracellular protease/amidase	-
Protease 2	3.4.-.-
Hydrogenase 3 large subunit C-terminal protease	-
Stomatin/prohibitin-family membrane protease subunit	-
MarP family serine protease	3.4.21.-
ATP-dependent zinc protease	3.4.24.-
Intracellular serine protease	3.4.21.-
Minor extracellular protease vpr	3.4.-.-
Protease PrtS	3.4.-.-
Protease synthase and sporulation protein PAI 2	3.4.-.-
Protease 1	3.4.-.-
ATP-dependent Clp protease proteolytic subunit 1	3.4.21.92
Putative cysteine protease YraA	-
Modulator of FtsH protease HflK	3.4.-.-
Zinc metalloprotease Rip1	3.4.24.-
Periplasmic serine endoprotease DegP	3.4.21.107
ATP-dependent Clp protease ATP-binding subunit ClpC1	3.4.-.-
Protease inhibitor I9 family protein	-
Prohead protease	3.4.-.-
ATP-dependent metalloprotease	3.4.24.-
Protease modulator HflK	3.-.-.-
Protease inhibitor Inh/omp19 family protein	-
AprI/Inh family metalloprotease inhibitor	3.4.-.-
DegQ family serine endoprotease	3.4.21.-
MprA protease, GlyGly-CTERM protein-sorting domain-containing form	3.4.-.-
YopT-type cysteine protease domain-containing protein	3.4.22.-
Putative glycoprotease GCP	-
Putative serine metalloprotease MprA	-
Putative type VII secretion-associated serine protease mycosin	-
Putative RIP metalloprotease RseP	-
Putative protease synthase and sporulation	-
Serine protease autotransporter toxin Sat	3.4.21.-
Metalloprotease LoiP	3.4.24.-
Serine protease autotransporter toxin SigA	3.4.21.-
Serine protease autotransporter toxin Pic	3.4.21.-
Cysteine protease staphopain B	3.4.22.-
CPBP family intramembrane metalloprotease SdpA	3.4.24.-
Serine protease SplF	3.4.21.-
Serine protease SplE	3.4.21.-
Serine protease SplD	3.4.21.-
Serine protease SplC	3.4.21.-

Serine protease SplB	3.4.21.-
Serine protease SplA	3.4.21.-
Cysteine protease staphopain A	3.4.22.-
CPBP family intramembrane metalloprotease SdpB	3.4.24.-
CPBP family intramembrane metalloprotease SdpC	3.4.24.-
Protease adaptor protein YjbH	-
ATP-dependent Clp protease ATP-binding subunit ClpL	3.4.21.92
Cysteine protease inhibitor staphostatin B	-
Xylanase	3.2.1.-
Endo-1,4-beta-xylanase	3.2.1.8
Glucuronoxylanase	3.2.1.136
1,4-beta-xylanase	3.2.1.8
Glucuronoarabinoxylan endo-1,4-beta-xylanase	3.2.1.136
Secreted endo-1,4-beta-xylanase	3.2.1.8
Putative xylanase	-
Putative xylanase/chitin deacetylase	-
Endo-1,4-beta-xylanase Z	3.2.1.8

Table S2. Gut microbiota isolates with amylase activity.

Aislado	Filo	Mejor coincidencia BLAST	REA
B51	Bacillota	*Bacillus mojavensis* [Beh2]	3,8±0,00
4.2	Pseudomonadota	*Burkholderia contaminans* [ILQ216]	3,7±0,00
B48	Bacillota	*Enterococcus durans* [DP2]	3,5±0,00
B20	Bacillota	*Enterococcus avium* [FDAARGOS_184]	3,4±0,26
B5	Pseudomonadota	*Escherichia coli* [RKH37_DCRUST]	3,4±0,00
B23	Bacillota	*Bacillus velezensis* [MOST-IAA]	3,1±0,29
B26	Bacillota	*Enterococcus gilvus* [3050]	3,1±0,06
92.2	Bacillota	*Bacillus amyloliquefaciens* [B1]	3,1±0,00
B4	Bacillota	*Bacillus siamensis* [VQ4]	3,1±0,00
B9	Bacillota	*Bacillus velezensis* [MOST-IAA]	3,1±0,00
B2	Pseudomonadota	*Escherichia coli* [RKH37_DCRUST]	3,1±0,00
B7, B8	Pseudomonadota	*Escherichia coli* [Wesam-23]	3,1±0,00
B56	Bacillota	*Bacillus subtilis* [LK3]	3,0±0,00
B40	Bacillota	*Bacillus velezensis* [YZF-2]	3,0±0,00
59.12	Bacillota	*Staphylococcus epidermidis* [LC5]	3,0±0,00
B37	Bacillota	*Bacillus velezensis* [T2]	2,9±0,16
B49	Bacillota	*Bacillus subtilis* [LK3]	2,9±0,00
34.1	Bacillota	*Bacillus nealsonii* [DSM 15077]	2,8±0,29
83.7.1	Bacillota	*Enterococcus faecium*	2,8±0,00
83.2.1	Bacillota	*Enterococcus faecium* [MJ1]	2,7±0,29
B16	Bacillota	*Bacillus velezensis* [T2]	2,7±0,14
57.1	Bacillota	*Staphylococcus epidermidis* [LC5]	2,7±0,12
92.9	Pseudomonadota	*Massilia haematophila*	2,7±0,00
92.8	Bacillota	*Siminovitchia fordii* [V28]	2,6±0,14
B25	Bacillota	*Enterococcus faecalis* [TMPC 63221]	2,6±0,12
B28	Pseudomonadota	*Escherichia coli* [Wesam-23]	2,6±0,00
53.3	Bacillota	*Enterococcus faecium* [MJ1]	2,5±0,00
77.8.1	Bacillota	*Bacillus rugosus* [Cq-55]	2,4±0,00
B3	Pseudomonadota	*Escherichia coli* [RKH37_DCRUST]	2,4±0,00
B32	Bacillota	*Bacillus thuringiensis* [S38]	2,3±0,00
B14	Bacillota	*Enterococcus faecalis* [QaAm-IRAQ-1]	2,3±0,00
B63	Bacillota	*Bacillus paralicheniformis* [HBUAS62636]	2,0±0,00
B15	Pseudomonadota	*Escherichia coli* [RKH37_DCRUST]	2,0±0,00
90.11	Bacillota	*Bacillus amyloliquefaciens* [SPa03NA]	1,9±0,00
20.3	Pseudomonadota	*Raoultella ornithinolytica* [P1]	1,9±0,00

REA: Relative enzymatic activity: Excellent: REA > 5, Good: REA > 2,0-5,0, Poor: REA < 2,0.

Table S3. Gut microbiota isolates with cellulase activity.

Isolate	Phylum	Best hit BLAST	REA
B20	*Bacillota*	*Enterococcus avium* [FDAARGOS_184]	3,3±0,00
B26	*Bacillota*	*Enterococcus gilvus* [3050]	3,1±0,17
B23	*Bacillota*	*Bacillus velezensis* [MOST-IAA]	3,1±0,00
B16	*Bacillota*	*Bacillus velezensis* [T2]	3,0±0,06
B9	*Bacillota*	*Bacillus velezensis* [MOST-IAA]	3,0±0,00
B5	*Pseudomonadota*	*Escherichia coli* [RKH37_DCRUST]	2,8±0,19
B15	*Pseudomonadota*	*Escherichia coli* [RKH37_DCRUST]	2,7±0,14
B2	*Pseudomonadota*	*Escherichia coli* [RKH37_DCRUST]	2,6±0,41
B4	*Bacillota*	*Bacillus siamensis* [VQ4]	2,6±0,00
57.1	*Bacillota*	*Staphylococcus epidermidis* [LC5]	2,5±0,50
83.7.1	*Bacillota*	*Enterococcus faecium*	2,5±0,33
B32	*Bacillota*	*Bacillus thuringiensis* [S38]	2,5±0,25
92.2	*Bacillota*	*Bacillus amyloliquefaciens* [B1]	2,5±0,12
B25	*Bacillota*	*Enterococcus faecalis* [TMPC 63221]	2,5±0,06
B3	*Pseudomonadota*	*Escherichia coli* [RKH37_DCRUST]	2,3±0,00
B8	*Pseudomonadota*	*Escherichia coli* [Wesam-23]	2,2±0,18
B14	*Bacillota*	*Enterococcus faecalis* [QaAm-IRAQ-1]	2,2±0,15
B40	*Bacillota*	*Bacillus velezensis* [YZF-2]	2,2±0,04
B7	*Pseudomonadota*	*Escherichia coli* [Wesam-23]	2,1±0,18
53.3	*Bacillota*	*Enterococcus faecium* [MJ1]	2,1±0,13
B37	*Bacillota*	*Bacillus velezensis* [T2]	2,1±0,08
4.2	*Pseudomonadota*	*Burkholderia contaminans* [ILQ216]	2,1±0,07
B63	*Bacillota*	*Bacillus paralicheniformis* [HBUAS62636]	2,0±0,43
92.8	*Bacillota*	*Siminovitchia fordii* [V28]	2,0±0,21
92.9	*Pseudomonadota*	*Massilia haematophila*	2,0±0,19
B56	*Bacillota*	*Bacillus subtilis* [LK3]	2,0±0,04
B48	*Bacillota*	*Enterococcus durans* [DP2]	2,0±0,00
83.2.1	*Bacillota*	*Enterococcus faecium* [MJ1]	2,0±0,00
59.12	*Bacillota*	*Staphylococcus epidermidis* [LC5]	2,0±0,00
B51	*Bacillota*	*Bacillus mojavensis* [Beh2]	1,9±0,12
1.2	*Actinomycetota*	*Micrococcus luteus* [NCCP 16831]	1,9±0,10
90.11	*Bacillota*	*Bacillus amyloliquefaciens* [SPa03NA]	1,9±0,00
34.1	*Bacillota*	*Bacillus nealsonii* [DSM 15077]	1,9±0,00
20.3	*Pseudomonadota*	*Raoultella ornithinolytica* [P1]	1,9±0,00
B49	*Bacillota*	*Bacillus subtilis* [LK3]	1,8±0,27
77.8.1	*Bacillota*	*Bacillus rugosus* [Cq-55]	1,8±0,00
30.1	*Actinomycetota*	*Microbacterium paraoxydans* [HZLJC2-1]	1,8±0,00
61.9	*Bacillota*	*Bacillus tropicus* [SAS-C1]	1,7±0,00
B28	*Pseudomonadota*	*Escherichia coli* [Wesam-23]	1,5±0,28
36.8	*Actinomycetota*	*Microbacterium paraoxydans* [CF36]	1,5±0,00

B6	*Bacillota*	*Lacticaseibacillus paracasei* [Lp02]	1,4±0,12

REA: Relative enzymatic activity: Excellent: REA > 5, Good: REA > 2,0-5,0, Poor: REA < 2,0.

Table S4. Gut microbiota isolates with inulinase activity.

Isolate	Phylum	Best hit BLAST	Growth
88.5	*Bacillota*	*Bacillus altitudinis* [Cq-36]	+
B62	*Bacillota*	*Bacillus altitudinis* [MY d]	+
92.2	*Bacillota*	*Bacillus amyloliquefaciens* [B1]	+
90.11	*Bacillota*	*Bacillus amyloliquefaciens* [SPa03NA]	+
B51	*Bacillota*	*Bacillus mojavensis* [Beh2]	+
34.1	*Bacillota*	*Bacillus nealsonii* [DSM 15077]	+
B63	*Bacillota*	*Bacillus paralicheniformis* [HBUAS62636]	+
77.5.2	*Bacillota*	*Bacillus pumilus* [Cq-35]	+
B39, B93	*Bacillota*	*Bacillus pumilus* [MUSRH05]	+
77.8.1	*Bacillota*	*Bacillus rugosus* [Cq-55]	+
B4	*Bacillota*	*Bacillus siamensis* [VQ4]	+
B49, B56	*Bacillota*	*Bacillus subtilis* [LK3]	+
B32, B34	*Bacillota*	*Bacillus thuringiensis* [S38]	+
B9, B23	*Bacillota*	*Bacillus velezensis* [MOST-IAA]	+
B16, B37	*Bacillota*	*Bacillus velezensis* [T2]	+
B40	*Bacillota*	*Bacillus velezensis* [YZF-2]	+
4.2	*Pseudomonadota*	*Burkholderia contaminans* [ILQ216]	+
B10	*Pseudomonadota*	*Enterobacter sp.* [APCB2]	+
B20	*Bacillota*	*Enterococcus avium* [FDAARGOS_184]	+
B41, B48	*Bacillota*	*Enterococcus durans* [DP2]	+
B11	*Bacillota*	*Enterococcus faecalis* [TMPC 63221]	+
83.7.1	*Bacillota*	*Enterococcus faecium*	+
83.2.1, 53.3	*Bacillota*	*Enterococcus faecium* [MJ1]	+
B26	*Bacillota*	*Enterococcus gilvus* [3050]	+
88.2, 83.8	*Bacillota*	*Enterococcus lactis* [Tw22]	+
83.3	*Pseudomonadota*	*Escherichia coli*	+
134.5, 57.5, 102.3, 102.1, 57.4, 102.2, 83.6.1	*Pseudomonadota*	*Escherichia coli* [EC12]	+
B64, B78	*Pseudomonadota*	*Escherichia coli* [M7]	+
B15, B2, B3, B5, B29, B33	*Pseudomonadota*	*Escherichia coli* [RKH37_DCRUST]	+
83.9	*Pseudomonadota*	*Escherichia coli* [ST865]	+
B7, B8, B28	*Pseudomonadota*	*Escherichia coli* [Wesam-23]	+
B6	*Bacillota*	*Lacticaseibacillus paracasei* [Lp02]	+

92.9	*Pseudomonadota*	*Massilia haematophila*	+
36.8	*Actinomycetota*	*Microbacterium paraoxydans* [CF36]	+
30.1	*Actinomycetota*	*Microbacterium paraoxydans* [HZLJC2-1]	+
1.2	*Actinomycetota*	*Micrococcus luteus* [NCCP 16831]	+
43.14	*Pseudomonadota*	*Pseudomonas synxantha* [27]	+
20.3	*Pseudomonadota*	*Raoultella ornithinolytica* [P1]	+
92.8	*Bacillota*	*Siminovitchia fordii* [V28]	+
59.12, 59.5, 57.1	*Bacillota*	*Staphylococcus epidermidis* [LC5]	+

Table S5. Gut microbiota isolates with nuclease activity.

Isolate	Phylum	Best hit BLAST	REA
B56	*Bacillota*	*Bacillus subtilis* [LK3]	2,5±0,00
B14	*Bacillota*	*Enterococcus faecalis* [QaAm-IRAQ-1]	2,2±0,01
B48	*Bacillota*	*Enterococcus durans* [DP2]	2,0±0,00
83.7.1	*Bacillota*	*Enterococcus faecium*	2,0±0,00
B7	*Pseudomonadota*	*Escherichia coli* [Wesam-23]	2,0±0,00
B55	*Bacillota*	*Staphylococcus sp.* [T45]	2,0±0,00
B8	*Pseudomonadota*	*Escherichia coli* [Wesam-23]	1,8±0,00
B20	*Bacillota*	*Enterococcus avium* [FDAARGOS_184]	1,7±0,38
B34	*Bacillota*	*Bacillus thuringiensis* [S38]	1,7±0,07
B42	*Bacillota*	*Bacillus sp.* [SD-37]	1,7±0,06
B15	*Pseudomonadota*	*Escherichia coli* [RKH37_DCRUST]	1,7±0,02
88.5	*Bacillota*	*Bacillus altitudinis* [Cq-36]	1,7±0,00
77.8.1	*Bacillota*	*Bacillus rugosus* [Cq-55]	1,7±0,00
83.2.1	*Bacillota*	*Enterococcus faecium* [MJ1]	1,6±0,23
B25	*Bacillota*	*Enterococcus faecalis* [TMPC 63221]	1,6±0,18
B38	*Bacillota*	*Bacillus altitudinis* [SA275C2]	1,6±0,08
B39	*Bacillota*	*Bacillus pumilus* [MUSRH05]	1,5±0,15
B26	*Bacillota*	*Enterococcus gilvus* [3050]	1,5±0,13
77.12	*Bacillota*	*Staphylococcus epidermidis* [OA162]	1,5±0,04
B62	*Bacillota*	*Bacillus altitudinis* [MY d]	1,5±0,00
B93	*Bacillota*	*Bacillus pumilus* [MUSRH05]	1,5±0,00
B51	*Bacillota*	*Bacillus mojavensis* [Beh2]	1,4±0,19
B49	*Bacillota*	*Bacillus subtilis* [LK3]	1,4±0,19
B63	*Bacillota*	*Bacillus paralicheniformis* [HBUAS62636]	1,3±0,00
B74, B75	*Bacillota*	*Bacillus thuringiensis* [S38]	1,3±0,00
B2, B3, B5	*Pseudomonadota*	*Escherichia coli* [RKH37_DCRUST]	1,3±0,00
92.8	*Bacillota*	*Siminovitchia fordii* [V28]	1,3±0,00
77.5.2	*Bacillota*	*Bacillus pumilus* [Cq-35]	1,2±0,16
B32	*Bacillota*	*Bacillus thuringiensis* [S38]	1,1±0,00
88.2	*Bacillota*	*Enterococcus lactis* [Tw22]	1,1±0,00
36.8	*Actinomycetota*	*Microbacterium paraoxydans* [CF36]	1,1±0,00
43.14	*Pseudomonadota*	*Pseudomonas synxantha* [27]	1,1±0,00

20.3	*Pseudomonadota*	*Raoultella ornithinolytica* [P1]	1,1±0,00
59.12, 57.1	*Bacillota*	*Staphylococcus epidermidis* [LC5]	1,1±0,00

REA: Relative enzymatic activity: Excellent: REA > 5, Good: REA > 2,0-5,0, Poor: REA < 2,0.

Table S6. Gut microbiota isolates with lipolitic activity.

Isolate	Phylum	Best hit BLAST	REA
30.1	*Actinomycetota*	*Microbacterium paraoxydans* [HZLJC2-1]	2,0±0,00
20.3	*Pseudomonadota*	*Raoultella ornithinolytica* [P1]	1,4±0,00
B29	*Pseudomonadota*	*Escherichia coli* [RKH37_DCRUST]	1,3±0,06
1.2	*Actinomycetota*	*Micrococcus luteus* [NCCP 16831]	1,3±0,05
90.11	*Bacillota*	*Bacillus amyloliquefaciens* [SPa03NA]	1,3±0,00
4.2	*Pseudomonadota*	*Burkholderia contaminans* [ILQ216]	1,3±0,00
57.5, 102.3, 102.1, 83.6.1	*Pseudomonadota*	*Escherichia coli* [EC12]	1,3±0,00
83.9	*Pseudomonadota*	*Escherichia coli* [ST865]	1,3±0,00
B34	*Bacillota*	*Bacillus thuringiensis* [S38]	1,2±0,08
B16	*Bacillota*	*Bacillus velezensis* [T2]	1,2±0,01
34.1	*Bacillota*	*Bacillus nealsonii* [DSM 15077]	1,2±0,00
B10	*Pseudomonadota*	*Enterobacter sp.* [APCB2]	1,2±0,00
B11	*Bacillota*	*Enterococcus faecalis* [TMPC 63221]	1,2±0,00
88.5	*Bacillota*	*Bacillus altitudinis* [Cq-36]	1,1±0,00
B62	*Bacillota*	*Bacillus altitudinis* [MY d]	1,1±0,00
B38	*Bacillota*	*Bacillus altitudinis* [SA275C2]	1,1±0,00
B51	*Bacillota*	*Bacillus mojavensis* [Beh2]	1,1±0,00
B63	*Bacillota*	*Bacillus paralicheniformis* [HBUAS62636]	1,1±0,00
77.5.2	*Bacillota*	*Bacillus pumilus* [Cq-35]	1,1±0,00
B39, B93	*Bacillota*	*Bacillus pumilus* [MUSRH05]	1,1±0,00
77.8.1	*Bacillota*	*Bacillus rugosus* [Cq-55]	1,1±0,00
B4	*Bacillota*	*Bacillus siamensis* [VQ4]	1,1±0,00
B42	*Bacillota*	*Bacillus sp.* [SD-37]	1,1±0,00
B49, B56	*Bacillota*	*Bacillus subtilis* [LK3]	1,1±0,00
B74, B75	*Bacillota*	*Bacillus thuringiensis* [S38]	1,1±0,00
B9, B23	*Bacillota*	*Bacillus velezensis* [MOST-IAA]	1,1±0,00
B37	*Bacillota*	*Bacillus velezensis* [T2]	1,1±0,00
B40	*Bacillota*	*Bacillus velezensis* [YZF-2]	1,1±0,00
88.2	*Bacillota*	*Enterococcus lactis* [Tw22]	1,1±0,00
134.5, 57.4, 102.2	*Pseudomonadota*	*Escherichia coli* [EC12]	1,1±0,00
B64, B78	*Pseudomonadota*	*Escherichia coli* [M7]	1,1±0,00
B2, B3, B5, B15	*Pseudomonadota*	*Escherichia coli* [RKH37_DCRUST]	1,1±0,00
B7, B8, B28	*Pseudomonadota*	*Escherichia coli* [Wesam-23]	1,1±0,00
B6	*Bacillota*	*Lacticaseibacillus paracasei* [Lp02]	1,1±0,00

36.8	*Actinomycetota*	*Microbacterium paraoxydans* [CF36]	1,1±0,00
92.8	*Bacillota*	*Siminovitchia fordii* [V28]	1,1±0,00

REA: Relative enzymatic activity: Excellent: REA > 5, Good: REA > 2,0-5,0, Poor: REA < 2,0.

Table S7. Gut microbiota isolates with proteolitic activity.

Isolate	Phylum	Best hit BLAST	REA
B26	*Bacillota*	*Enterococcus gilvus* [3050]	2,5±0,08
B2, B3, B5	*Pseudomonadota*	*Escherichia coli* [RKH37_DCRUST]	2,5±0,00
B74	*Bacillota*	*Bacillus thuringiensis* [S38]	2,5±0,00
B9	*Bacillota*	*Bacillus velezensis* [MOST-IAA]	2,5±0,00
B8	*Pseudomonadota*	*Escherichia coli* [Wesam-23]	2,5±0,00
53.3	*Bacillota*	*Enterococcus faecium* [MJ1]	2,4±0,00
B49	*Bacillota*	*Bacillus subtilis* [LK3]	2,3±0,18
B75	*Bacillota*	*Bacillus thuringiensis* [S38]	2,3±0,10
92.2	*Bacillota*	*Bacillus amyloliquefaciens* [B1]	2,3±0,00
B40	*Bacillota*	*Bacillus velezensis* [YZF-2]	2,3±0,00
92.9	*Pseudomonadota*	*Massilia haematophila*	2,2±0,06
B37	*Bacillota*	*Bacillus velezensis* [T2]	2,2±0,00
20.3	*Pseudomonadota*	*Raoultella ornithinolytica* [P1]	2,2±0,00
B51	*Bacillota*	*Bacillus mojavensis* [Beh2]	2,1±0,05
90.11	*Bacillota*	*Bacillus amyloliquefaciens* [SPa03NA]	2,1±0,00
B20	*Bacillota*	*Enterococcus avium* [FDAARGOS_184]	2,1±0,00
34.1	*Bacillota*	*Bacillus nealsonii* [DSM 15077]	2,0±0,04
4.2	*Pseudomonadota*	*Burkholderia contaminans* [ILQ216]	2,0±0,04
B7	*Pseudomonadota*	*Escherichia coli* [Wesam-23]	2,0±0,00
B16	*Bacillota*	*Bacillus velezensis* [T2]	1,9±0,19
B62	*Bacillota*	*Bacillus altitudinis* [MY d]	1,9±0,12
B38	*Bacillota*	*Bacillus altitudinis* [SA275C2]	1,9±0,00
B39	*Bacillota*	*Bacillus pumilus* [MUSRH05]	1,9±0,00
B56	*Bacillota*	*Bacillus subtilis* [LK3]	1,9±0,00
92.8	*Bacillota*	*Siminovitchia fordii* [V28]	1,9±0,00
77.5.2	*Bacillota*	*Bacillus pumilus* [Cq-35]	1,8±0,04
B34	*Bacillota*	*Bacillus thuringiensis* [S38]	1,8±0,04
B23	*Bacillota*	*Bacillus velezensis* [MOST-IAA]	1,8±0,03
B93	*Bacillota*	*Bacillus pumilus* [MUSRH05]	1,8±0,00
B42	*Bacillota*	*Bacillus sp.* [SD-37]	1,8±0,00
59.12	*Bacillota*	*Staphylococcus epidermidis* [LC5]	1,8±0,00
61.9	*Bacillota*	*Bacillus tropicus* [SAS-C1]	1,7±0,16
B15	*Pseudomonadota*	*Escherichia coli* [RKH37_DCRUST]	1,7±0,10
62.5	*Bacillota*	*Enterococcus faecalis* [LA-11]	1,7±0,00
88.5	*Bacillota*	*Bacillus altitudinis* [Cq-36]	1,6±0,00
B63	*Bacillota*	*Bacillus paralicheniformis* [HBUAS62636]	1,5±0,08
1.2	*Actinomycetota*	*Micrococcus luteus* [NCCP 16831]	1,5±0,03

30.1	*Actinomycetota*	*Microbacterium paraoxydans* [HZLJC2-1]	1,4±0,08

REA: Relative enzymatic activity: Excellent: REA > 5, Good: REA > 2,0-5,0, Poor: REA < 2,0.

Table S8. Gut microbiota isolates with gelatinase activity.

Isolate	Phylum	Best hit BLAST	REA
B29	*Pseudomonadota*	*Escherichia coli* [RKH37_DCRUST]	4,8±0,00
4.2	*Pseudomonadota*	*Burkholderia contaminans* [ILQ216]	4,4±0,00
92.9	*Pseudomonadota*	*Massilia haematophila*	3,9±0,26
B20	*Bacillota*	*Enterococcus avium* [FDAARGOS_184]	3,9±0,00
B51	*Bacillota*	*Bacillus mojavensis* [Beh2]	3,8±0,00
62.4	*Bacillota*	*Enterococcus faecalis* [LA-11]	3,8±0,00
B26	*Bacillota*	*Enterococcus gilvus* [3050]	3,8±0,00
59.12	*Bacillota*	*Staphylococcus epidermidis* [LC5]	3,8±0,00
34.1	*Bacillota*	*Bacillus nealsonii* [DSM 15077]	3,7±0,00
B9	*Bacillota*	*Bacillus velezensis* [MOST-IAA]	3,6±0,30
B40	*Bacillota*	*Bacillus velezensis* [YZF-2]	3,6±0,10
92.8	*Bacillota*	*Siminovitchia fordii* [V28]	3,5±0,13
83.7.1	*Bacillota*	*Enterococcus faecium*	3,5±0,00
B37	*Bacillota*	*Bacillus velezensis* [T2]	3,4±0,21
B14	*Bacillota*	*Enterococcus faecalis* [QaAm-IRAQ-1]	3,4±0,00
B16	*Bacillota*	*Bacillus velezensis* [T2]	3,3±0,06
B42	*Bacillota*	*Bacillus sp.* [SD-37]	3,3±0,02
88.5	*Bacillota*	*Bacillus altitudinis* [Cq-36]	3,3±0,00
B48	*Bacillota*	*Enterococcus durans* [DP2]	3,3±0,00
20.3	*Pseudomonadota*	*Raoultella ornithinolytica* [P1]	3,3±0,00
B4	*Bacillota*	*Bacillus siamensis* [VQ4]	3,2±0,49
B2	*Pseudomonadota*	*Escherichia coli* [RKH37_DCRUST]	3,2±0,48
B39	*Bacillota*	*Bacillus pumilus* [MUSRH05]	3,2±0,23
B23	*Bacillota*	*Bacillus velezensis* [MOST-IAA]	3,2±0,06
B3	*Pseudomonadota*	*Escherichia coli* [RKH37_DCRUST]	3,1±0,56
B38	*Bacillota*	*Bacillus altitudinis* [SA275C2]	3,1±0,37
B5	*Pseudomonadota*	*Escherichia coli* [RKH37_DCRUST]	3,1±0,27
B93	*Bacillota*	*Bacillus pumilus* [MUSRH05]	3,1±0,00
B8	*Pseudomonadota*	*Escherichia coli* [Wesam-23]	3,0±0,22
77.5.2	*Bacillota*	*Bacillus pumilus* [Cq-35]	3,0±0,00
B49	*Bacillota*	*Bacillus subtilis* [LK3]	3,0±0,00
B15	*Pseudomonadota*	*Escherichia coli* [RKH37_DCRUST]	3,0±0,00
57.1	*Bacillota*	*Staphylococcus epidermidis* [LC5]	3,0±0,00
B63	*Bacillota*	*Bacillus paralicheniformis* [HBUAS62636]	2,9±0,29
B56	*Bacillota*	*Bacillus subtilis* [LK3]	2,8±0,31
83.2.1	*Bacillota*	*Enterococcus faecium* [MJ1]	2,8±0,00
B7	*Pseudomonadota*	*Escherichia coli* [Wesam-23]	2,7±1,13
B32	*Bacillota*	*Bacillus thuringiensis* [S38]	2,7±0,21

92.2	*Bacillota*	*Bacillus amyloliquefaciens* [B1]	2,7±0,00
77.8.1	*Bacillota*	*Bacillus rugosus* [Cq-55]	2,7±0,00
B6	*Bacillota*	*Lacticaseibacillus paracasei* [Lp02]	2,6±0,16
90.11	*Bacillota*	*Bacillus amyloliquefaciens* [SPa03NA]	2,6±0,13
B62	*Bacillota*	*Bacillus altitudinis* [MY d]	2,5±0,00
B25	*Bacillota*	*Enterococcus faecalis* [TMPC 63221]	2,5±0,00
B34, B74, B75	*Bacillota*	*Bacillus thuringiensis* [S38]	2,3±0,40
62.5	*Bacillota*	*Enterococcus faecalis* [LA-11]	2,2±0,07
B28	*Pseudomonadota*	*Escherichia coli* [Wesam-23]	1,9±0,60
B33	*Pseudomonadota*	*Escherichia coli* [RKH37_DCRUST]	1,8±0,13
36.8	*Actinomycetota*	*Microbacterium paraoxydans* [CF36]	1,6±0,22
61.9	*Bacillota*	*Bacillus tropicus* [SAS-C1]	1,4±0,07

REA: Relative enzymatic activity: Excellent: REA > 5, Good: REA > 2,0-5,0, Poor: REA < 2,0.
